# Size Distribution of Zinc Oxide Nanoparticles Depending on the Temperature of Electrochemical Synthesis

**DOI:** 10.3390/ma18020458

**Published:** 2025-01-20

**Authors:** Michał Hajos, Maria Starowicz, Beata Brzychczyk, Grzegorz Basista, Sławomir Francik

**Affiliations:** 1 Department of Mechanical Engineering and Agrophysics, Faculty of Production and Power Engineering, University of Agriculture in Krakow, Balicka Street 116 B, 30-149 Krakow, Poland; beata.brzychczyk@urk.edu.pl (B.B.); grzegorz.basista@urk.edu.pl (G.B.); 2 Department of Chemistry and Corrosion of Metals, Faculty of Foundry Engineering, AGH University of Krakow, Reymonta Street 23, 30-059 Krakow, Poland; mariast@agh.edu.pl

**Keywords:** nanoparticles, zinc oxide (ZnO), nanoparticle size distribution, nanoparticle electrochemical synthesis

## Abstract

One of the methods for obtaining zinc oxide nanoparticles (ZnO NPs) is electrochemical synthesis. In this study, the anodic dissolution process of metallic zinc in alcohol solutions of LiCl was used to synthesize ZnO NPs. The products were obtained as colloidal suspensions in an electrolyte solution. Due to the small size and ionic nature of the zinc oxide molecule, colloidal nanoparticles tend to cluster into larger groupings, so the size of nanoparticles in solutions will differ from the size of nanoparticles observed in ZnO powders after solvent evaporation. The main goal of this research is to investigate the influence of the temperature of synthesis and the kind of alcohol on the size of ZnO NP micelles. Nanocrystals of zinc oxide were obtained in all tested alcohols: methanol, ethanol, and 1-propanol. The particle size was determined using the Dynamic Light Scattering (DLS) method. It was observed that the particles synthesized in methanol were the largest, followed by smaller particles in ethanol, while the smallest particles were obtained in 1-propanol. Additionally, the particles obtained in ethanol were the most uniform in size, showing the highest level of size homogeneity.

## 1. Introduction

Nanotechnology is a rapidly growing field of science that involves the design, manufacture, and use of materials, devices, and systems with sizes ranging from 1 nm to 100 nm [1,2]. The extremely small size of nanostructures means that the newly obtained materials have different properties compared to their corresponding macroscale materials. This unique behavior has driven significant interest in nanomaterials due to their exceptional electrical, optical, and surface properties [3,4].

Among nanoparticles, metal and metal oxide nanoparticles, including zinc oxide nanoparticles (ZnO NPs), hold a prominent position [5,6].

Zinc oxide nanoparticles find applications in many sectors of the economy [4,7,8]. ZnO NPs are used in fields such as optics, electronics, food packaging, and medicine [7]. Scherzad et al. even stated that ZnO NPs are among the most frequently used nanomaterials in industrial applications (e.g., rubber production, cement additive, ingredients of pigments and paints, UV filters, etc.) [4]. ZnO NPs are also used in the production of gas sensors, biological sensors, cosmetics, ceramics, rubber, optical devices, and solar cells (gas sensors, biosensors, cosmetics, ceramics, rubber, optical devices, solar cells) [9].

Zinc oxide is particularly valued for its antibacterial and wound-healing properties [10,11], making it a key ingredient in medicinal and pharmaceutical products, such as poultices and ointments. An example is the composites based on gelatin nanofibers and ZnO nanoparticles (composites based on gelatin nanofibers and ZnO nanoparticles) developed by Babayewska et al., showing antibacterial activity against Gram-positive and Gram-negative bacteria [12]. It can also serve as a carrier for active substances in advanced modern drug delivery systems [13,14]. Biological applications include research on the use of ZnO NPs for protein detection, biological imaging, drug delivery, and combating cancer cells [14]. Research is being carried out on the use of ZnO NPs in dentistry [15] and the creation of biodegradable films used in tissue engineering and wound treatment (tissue engineering, wound treatment) [16]. Thus, ZnO nanoparticles show great potential in medical applications [17].

The antibacterial properties of zinc oxide are also used in the textile industry, where zinc oxide is used, like silver ions, in the finishing of fabrics, providing protection against bacteria and fungi [18,19,20]. Additionally, its use in food packaging enhances microbial resistance, protecting food from bacterial and fungal contamination [14,21,22].

ZnO NPs are also used in agriculture [17]. The antibacterial and antifungal properties of zinc oxide [22] make it useful in the production of plant protection products, fertilizers, and seed protection preparations. As a micronutrient, it supports plant growth and, unlike silver ions, poses no risk to soil microorganisms.

ZnO NPs are also used to remove pollutants in water systems (photocatalytic degradation of organic dyes) [23].

Zinc oxide is also widely used for its electrical properties. As an n-type semiconductor, it is used in the production of transistors, LEDs, and solar cells [24,25,26], as well as gas detectors [27].

The optical properties of zinc oxide make it a useful material in cosmetics. Due to its effective ultraviolet (UV) radiation absorption, it is used in sunscreen formulations [4,28,29]. Due to the ability to absorb ultraviolet radiation, it is also used as an additive in the production of UV-protective paints and varnishes [30,31], where ZnO not only provides UV shielding but also increases the durability and microbial resistance of the coatings.

Many methods have been developed for the production of nanoparticle materials. Among these, there are two main groups of processes. One is based on the fragmentation of materials (top-down method), and the other is an incremental method (bottom-up method) [8,9,32]. One of the methods for obtaining zinc oxide nanoparticles that integrate elements of both approaches is the electrochemical synthesis method developed by Prof. Stypuła and her associates. This technique involves the initial dissolution of metallic zinc and the subsequent formation of zinc oxide nanoparticles as a result of processes taking place in the electrolyte [33].

## 2. Materials and Methods

### 2.1. Materials

The chemicals used in the investigation were methanol (99.9%), ethanol (99.8%), and 1-propanol (99.5%), all produced by Avantor Performance Materials Poland S.A. Lithium chloride (LiCl ≥ 99.0%) was produced by Merc and zinc foil (99.99% Goodfellow). All the reagents were used as received without further purification.

### 2.2. Synthesis of ZnO Nanoparticles (ZnO NPs)

The electrochemical production of ZnO NPs was performed in a one-compartment glass electrochemical cell using a simple system of three zinc anodes in a 50 × 10 × 0.2 mm^3^ plate and two zinc cathodes with the same shape and size. The cathodes were situated halfway between the anodes. The distance between neighboring electrodes was 20 mm. The synthesis of ZnO NPs was performed at the temperatures of 20, 30, 40, and 50 °C with a current density of 0.5 mAcm^−2^. In order to maintain a constant temperature, the electrochemical cell was thermostated in a water bath. The polarization process was carried out in a solution of 0.05 M LiCl (alcohol +5% vol. H_2_O). Simple alcohols were used: methanol, ethanol, and 1-propanol. A detailed description of the synthesis is presented in our previous publications [33,34].

### 2.3. Methods

Zinc oxide nanoparticles were produced using the electrochemical dissolution of metallic zinc [33]. The process uses zinc electrodes immersed in an alcoholic salt solution with water addition. The electrodes are connected to an external power source, causing the electrode to act as the anode and to be etched and dissolved. Zn^2+^ metal ions enter the solution and react with alcohol molecules to form alcoholates, which then undergo hydrolysis under the influence of water, producing zinc oxide. During the process, the solution is blown with inert gas (argon) to deoxidize the electrolyte. Additionally, the gas stream mixes the solution, supporting the transport of ions, especially in the space near the electrode.

The process results in a suspension—a colloidal solution of zinc oxide nanoparticles. The formation of zinc oxide nanoparticles depends on a number of parameters, primarily the type of alcohol solvent used, the type of salt forming the electrolyte, the salt concentration, and the quantitative addition of water. The detailed influence of these parameters on the electrochemical synthesis of zinc oxide nanoparticles was described in the papers [34,35].

Based on the experimental results, the optimal process parameters were determined. The formation of zinc oxide nanoparticles as the final product of the process has been confirmed by a number of analytical methods. The tests were carried out on synthesis products in the form of colloidal solutions and nanopowders obtained after evaporation of the solvent.

The determination of the chemical composition of the deposit was realized by X-ray diffraction (XRD) with a Philips PW-3710 X’PERT diffractometer using Cu-Ka radiation. The morphology of the deposit was studied by transmission electron microscopy (TEM) using a Phillips CM20 TWIN microscope operating at 200 kV.

Particle size distribution in suspension was determined by dynamic laser light scattering (DLS) using a NanoSizer-ZS (Malvern Inc. Malvern Instruments Ltd, Malvern, Worcestershire, United Kingdom). The diameter of the particles determined as a result of the measurement is their hydrodynamic diameter, i.e., the diameter of a hypothetical rigid sphere diffusing at the same speed as the diffusing measured particle.

As a result of the calculations based on scattered light intensity values, a graph of the particle size distribution—the percentage of particles in the sample depending on their size—is obtained as a result of the particle size measurement.

To determine the size of zinc oxide nanoparticles in solutions, before each measurement, the tested samples were placed in an ultrasonic bath to break up the forming particle agglomerates.

The Zetasizer Software (Zetasizer Series Software Suite v. 6.01), which controls the NanoSizer-ZS, allows the number of measurements taken per sample to be manually set. A higher number of repetitions of measurement per sample increases the precision of results for samples with high variability. Most commonly, 3 to 5 measurements are taken. The typical time for one measurement, or one data acquisition sequence, takes 1–2 minutes. In automatic operating mode, the instrument decides on the basis of its calculations how long it takes to obtain a reliable result.

The examination of each sample included five consecutive measurements of the same colloidal suspension.

## 3. Results

**Figure 1:**
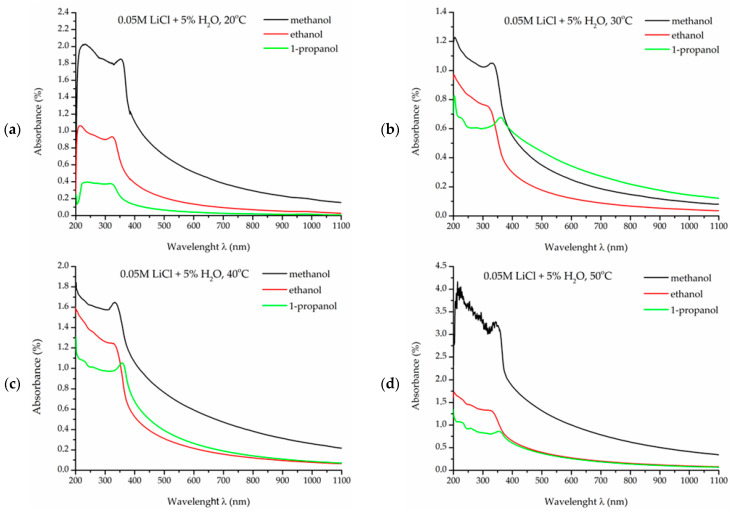
UV-vis spectra of ZnO nanoparticles obtained in 0.05 M LiCl solutions with 5% H_2_O in alcohol solvents at temperatures: (**a**) 20 °C, (**b**) 30 °C, (**c**) 40 °C, (**d**) 50 °C.

Preliminary analyses of colloidal solutions with alcohol solvents, methanol, ethanol, and 1-propanol were conducted using the UV-vis (ultraviolet–visible light spectroscopy) method. The results showed the presence of zinc oxide in the tested suspensions. In the obtained spectra, a peak characteristic of ZnO was observed in the wavelengths (λ) ranging between 350 and 370 nm (Figure 1a–d), which is consistent with literature data [36,37].

The final products of the synthesis process were also tested after the evaporation of the solvents. Analyses of the powders using SEM-EDX (scanning electron microscopy with energy dispersive X-ray analysis), X-ray diffraction (XRD), and electron diffraction methods showed that the tested powders were zinc oxide but only in the case of samples synthesized in solutions with methanol and ethanol solvents. Products obtained in 1-propanol environments, in addition to zinc oxide, also contained zinc salts and hydroxy salts.

**Figure 2:**
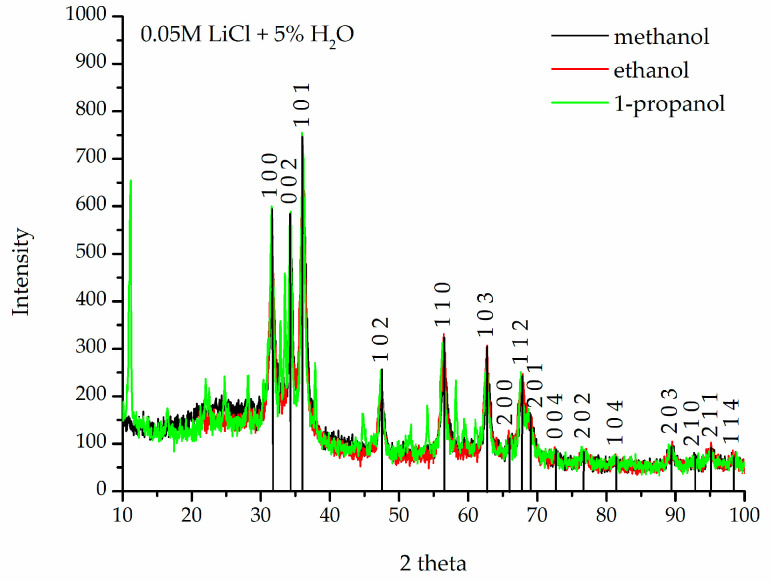
X-ray diffraction (XRD) spectra of ZnO nanoparticles obtained in 0.05 M LiCl alcohol solutions with 5% H_2_O in alcohol solvents at 20 °C.

Figure 2 shows the results of XRD analysis of the nanopowders obtained in 0.05 M LiCl solution with 5% vol. water. The spectra show peaks characteristic of crystalline zinc oxide, recorded at corresponding values of angle 2 theta. Each peak corresponds to a plane of the ZnO crystal lattice identified by Miller’s plane indices. The fit is consistent with the pattern for ZnO [38].

The structure of zinc oxide nanopowders (after evaporation of the solvent) was determined based on TEM and electron diffraction studies. Based on these analyses, the optimal parameters for the synthesis of nanoparticles were selected during the anodic dissolution of zinc as a galvanostatic process in alcoholic salt solutions.

The measurement of particle size in colloidal solutions was carried out for samples obtained in methanol, ethanol, and 1-propanol solutions with a lithium chloride concentration of 0.05 mol/dm^3^ (0.05 M LiCl), with the addition of 5% water by volume. An additional parameter that differentiated the samples was the temperature at which the synthesis process was conducted. The temperature range used was 20, 30, 40, and 50 °C from ambient temperature to the value below the boiling point of methanol, the first alcohol in the series.

An increase in the temperature of the electrolyte causes an increase in the kinetic energy of the molecules, and this leads to an increase in the rate of electrode reactions. Higher temperatures reduce the activation overvoltage, facilitating the detachment of zinc cations from the electrode surface and their subsequent migration into the solution. Increased temperature also enhances the mobility of the ions (Zn^2+^, Cl^−^, H^+^, OH^−^) present in the electrolyte, resulting in higher conductivity and lower cell resistance. As a result, charge transport is facilitated, and zinc dissolution becomes more efficient. The aforementioned increased mobility of ions in the electrolyte also means an increased number of active collisions, stimulating the subsequent reactions taking place in the electrolyte.

**Figure 3:**
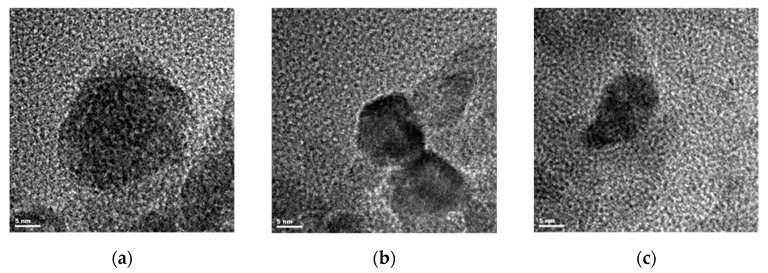
TEM images of zinc oxide nanoparticles synthesized in 0.05 M LiCl alcohol solutions with the addition of 5% water by volume: (**a**) methanol; (**b**) ethanol; (**c**) 1-propanol.

The size of the zinc oxide nanoparticles obtained by the electrochemical method was determined using TEM microscopic technique (Figure 3). These were observations of single particles or their small groups after the solvent had evaporated. The images shown in Figure 3 show zinc oxide nanoparticles obtained in salt solutions with alcohol solvents, with the addition of water.

Visible ZnO nanoparticles obtained in methanol solution showed a large size variation ranging from 10–20 nm to 30–50 nm.

In the ethanol environment, zinc oxide nanoparticles were more fragmented, and their size was more uniform.

Nanoparticles of the smallest sizes were obtained in 1-propanol, but the purity of the final synthesis product was a problem. In addition to zinc oxide nanoparticles, other phases were also present in the solution.

However, the above observations do not provide information on the size and behavior of zinc oxide nanoparticles in the form of a colloidal suspension, i.e., immediately after synthesis, in the form in which they can be stored or used [5].

### 3.1. Nanoparticles Electrochemical Synthesis at 20 °C

The zinc oxide nanoparticle size distribution figure shows the scattered light intensity values corresponding to the number and size of the recorded nanoparticles. The graphs show the course of the next five recorded nanoparticle size distributions.

**Figure 4:**
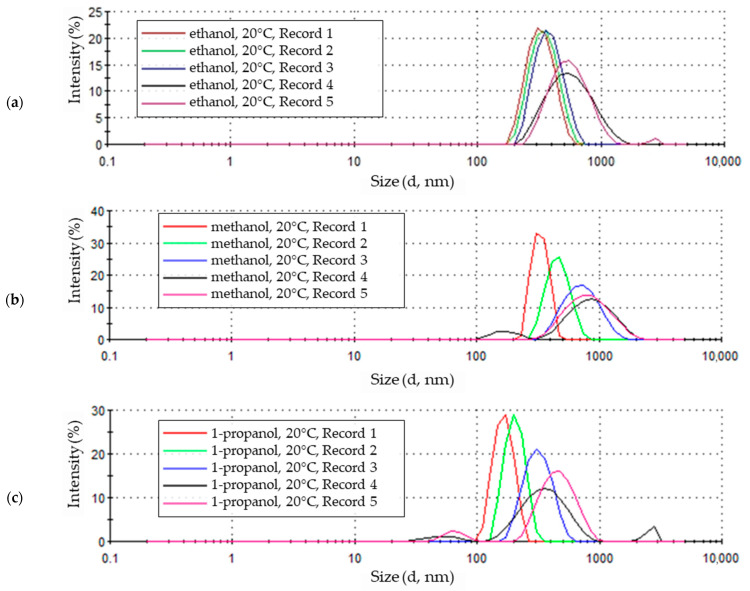
Zinc oxide nanoparticle size distribution by intensity in 0.05M LiCl alcoholic solutions with 5% vol. H_2_O at 20 °C in (**a**) methanol (average particle size 641.9 nm), (**b**) ethanol (average particle size 469.9 nm), (**c**) 1-propanol (average particle size 392.5 nm).

Figure 4a shows the size distribution intensity of zinc oxide nanoparticles synthesized in a 0.05 molar LiCl solution with a methanol solvent with the addition of 5% by volume of water at a temperature of 20 °C

The first of a series of five measurements shows a clearly defined peak with an intensity exceeding 30%, covering particle sizes ranging from 200 to 500 nm (Figure 4a). The peak corresponding to the second sample measurement is also well-defined but is characterized by a lower intensity, around 25%. It also represents particles with larger sizes, from approximately 250 to 900 nm. The next three signals recorded for this sample appear as peaks with decreasing intensity—down to approximately 12–15%, covering increasingly larger particle sizes, with a simultaneous shift of the maximum of the peaks towards larger sizes, from approximately 700 to 900 nm.

The size distribution of the products synthesized at a temperature of 20 °C in an ethanol medium is shown in Figure 4b. The peaks of the first three sample measurements are very clustered and show similar intensity at about 20% and maxima corresponding to particle sizes of 300–330 nm. There is a slight shift of each subsequent waveform, indicating a larger range of measured particle sizes from about 170 nm to 800 nm. The last two traces for the sample obtained at 20 °C in an ethanol solvent are also clustered, indicating a similar particle size, ranging from about 200 nm to about 1200 nm. However, the peak maxima in these traces reach a lower intensity, about 12–15%, for particle sizes around 520 nm.

In the last of the analyzed solutions—0.05 M LiCl with a 1-propanol solvent and 5% water by volume (Figure 4c)—the character of subsequent measurements of the size of ZnO nanoparticles obtained at 20 °C is similar to those in solutions with methanol and ethanol solvents. The results of the first two waveforms are represented by clear peaks with an intensity reaching approximately 30% for nanoparticle sizes of about 150 nm and about 200 nm, with a size range from about 100 nm to about 260 nm. The second peak is slightly shifted toward larger particle sizes, from about 120 nm to about 340 nm. The waveforms of the remaining three sample measurements, similar to the methanol solution, show a shift of the maxima of subsequent peaks towards larger particle sizes from about 300 nm to about 500 nm. Additionally, in the case of the last two sample records, the lack of uniformity in the size of the tested particles is visible, represented by more than one peak of the waveform.

**Figure 5:**
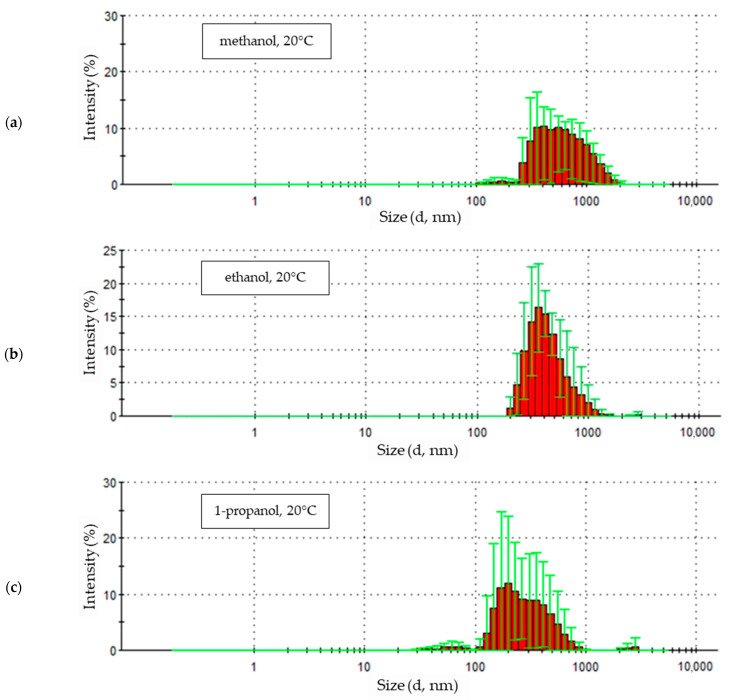
Statistical size distribution of ZnO nanoparticles synthesized in 0.05 M LiCl alcoholic solutions with 5% vol. H_2_O at 20 °C (five measurements) in (**a**) methanol, (**b**) ethanol, (**c**) 1-propanol.

The graphs below (Figure 5) show the statistical size distribution of ZnO nanoparticles synthesized in solutions with alcohol solvents at 20 °C. They were created based on a series of five measurements carried out for each sample, as shown in the previous charts (Figure 4). The signal intensity for each measured particle diameter value corresponds to the share of a given fraction in the total number of particles detected by the device’s detector. The number of particles of a given diameter occurring in the tested sample was given regardless of which of the five measurements it was determined in.

Nanoparticles obtained in methanol and 1-propanol solutions at 20 °C show a very wide range of particle size distribution (Figure 5). The size of particles obtained in a methanol medium ranges from about 100 nm to 2000 nm, and in the case of a 1-propanol solution, from about 30 nm to 860 nm, with a slight separation of a phase with a diameter in the range of 2000 nm to 2800 nm. The signal intensity for the particle diameters was 10.3% for a sample from the methanol solution, with a particle size of 412.5 nm. For particles synthesized in a 1-propanol environment, the maximum intensity reached 12% for particles that were almost exactly half the size, with a diameter of 198.0 nm. The given maximum signal intensity indicates the diameter of the largest particle size fraction in a given sample.

The particle size distributions from methanol (Figure 5a) and 1-propanol (Figure 5c) solutions are similar. In both cases, a small fraction of particles of small diameter was observed (229.3 nm and 265.6 nm for methanol and 127.5 nm and 147.7 nm for 1-propanol). These distributions are dominated by signals of similar intensity, indicating the presence in the samples of similar numbers of particles of increasingly larger sizes, the quantitative share of which decreases gently as they reach their maximum sizes.

The statistical size distribution of ZnO particles produced at 20 °C in an ethanol solution with the addition of 5% water by volume (Figure 5b) differs from the results obtained for samples synthesized in other electrolytes with methanol and 1-propanol solvents. The maximum signal intensity values reach higher values, up to 16.3%, which indicates a larger quantitative share of particles with a specific diameter in the sample (265.6 nm). This means that the particles formed in the ethanol solvent solution were much more uniform in size.

The statistical distribution of particles obtained in the ethanol environment, similarly to the other alcoholic solvents used, shows a small share of particles, with the smallest diameters (198.0 nm, 229.3 nm, and 265.6 nm) and the formation of increasingly smaller amounts of particles in the area of the largest diameters.

### 3.2. Nanoparticles Electrochemical Synthesis at 30 °C

**Figure 6:**
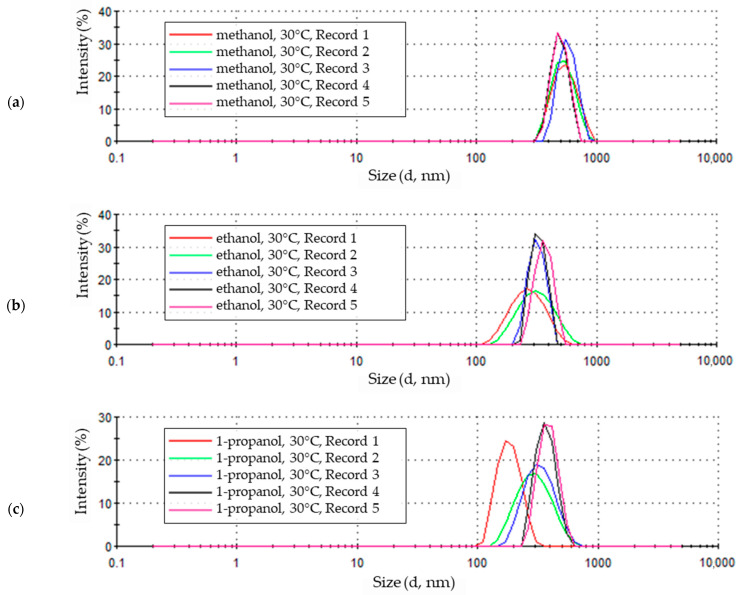
Zinc oxide nanoparticle size distribution by intensity in 0.05 M LiCl alcoholic solutions with 5% vol. H_2_O at 30 °C in (**a**) methanol (average particle size 682.8 nm), (**b**) ethanol (average particle size 362.9 nm), (**c**) 1-propanol (average particle size 354.2 nm).

For the reaction environment at a temperature of 30 °C, the intensity of subsequent recorded signals ranges from approximately 23% to 32%. The lowest values (approximately 23% and 25%) were observed in the first two measurements, and their maxima are not clearly visible. The remaining three measurements in the series of five have clearly defined maxima reaching the highest intensity (Figure 6a). This series of five consecutive signals recorded during the sample test indicates that in the initial phase of the measurement (first and second signal), ZnO particle sizes ranged from 300 nm to 1000 nm, with a maximum value of approximately 500–600 nm. The last three signals, with the highest intensity, indicate a larger number of particles with smaller size differences, as evidenced by the clearly marked shape of the peak maximum, which shifted towards smaller particle sizes (a larger number of smaller particles at the end of the measurement may indicate the gravitational fall of the largest and heaviest of them).

The size distribution of zinc oxide particles formed at a temperature of 30 °C in ethanolic solution is shown in Figure 6b. The difference between the signals for the analyzed sample is clearly visible. The first two are distinguished by a lower intensity of 16–17% (of refracted light) and cover the particle size range from just over 100 nm to about 600 nm for the first measurement and from about 120 nm to almost 800 nm in the second measurement. The last three results take the form of peaks with clearly defined maxima with twice the value from about 32% to about 34%, with the penultimate peak showing the highest value. The two peaks from the third and fourth measurements cover the particle size range from about 200–220 nm to about 500 nm. The last measurement covers the size of particles ranging from 220 nm to 530 nm.

The maximum intensity value of each of the subsequent five sample measurements is shifted towards larger particle sizes. This indicates that in the initial phase of measurement, particles with a smaller size of the dominant fraction are recorded. Combined with the low peak intensity and a wide range of particle sizes, this indicates the presence of particles of various sizes in the sample. In the final stage of the test, the intensity of the peaks increases and remains at a similar level, and this is accompanied by a narrowing of the particle size range in the solution. This indicates the detection of larger particles of less varying sizes.

The measurement results of the nanoparticle sizes obtained in a LiCl 1-propanol solution with the addition of 5% water by volume at a temperature of 30 °C are shown in Figure 6c. The first of a series of five measurements made on the sample has the form of a well-developed peak with a maximum intensity of approximately 25% for a particle size of approximately 160 nm. This peak covers the particle sizes between about 100 nm and about 340 nm. The next two measurements are peaks of lower intensity, with maxima of approximately 17–19%, shifted towards larger particle sizes of approximately 300 nm. The lower intensity peaks also cover a larger range of particle sizes, from approximately 120–130 nm to 700–800 nm. The last two measurements of the tested sample again take the form of well-developed peaks, with a maximum intensity higher than the initial peaks, reaching a value of about 28% at a particle size of about 400 nm. The highest intensity peaks also cover a narrower particle size range from about 220 nm to about 620 nm.

The changes in the shape and position of the measurement signals indicate changes in the colloid structure during the measurement. The initially recorded clear, high-intensity peak indicates the presence of a large number of particles of little diversity in size. It corresponds to the initial condition of the sample subjected to an ultrasonic treatment before testing. In subsequent measurements, the intensity of the peaks decreases, the maximum of the peaks shifts towards larger particle sizes, and the size range of the identified particles also increases. This is the stage in which the size of particles in the sample changes, as evidenced by gently outlined intensity maxima and a wide range of particle sizes. Based on the results of the last two measurements, it can be concluded that the changes taking place in the colloidal solution are over, as the last two peaks largely overlap. They achieve similar maximum intensity for similar particle sizes and contain a similar range of particle sizes. This means that in the final phase of the measurement, the sample contains more uniform particles with larger sizes than initially.

**Figure 7:**
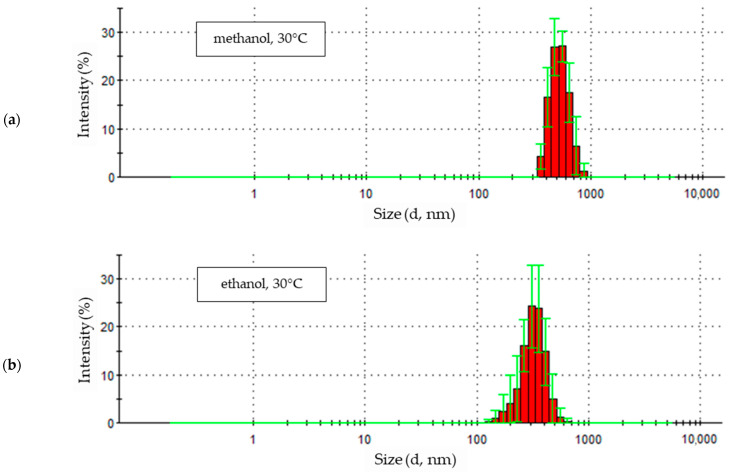

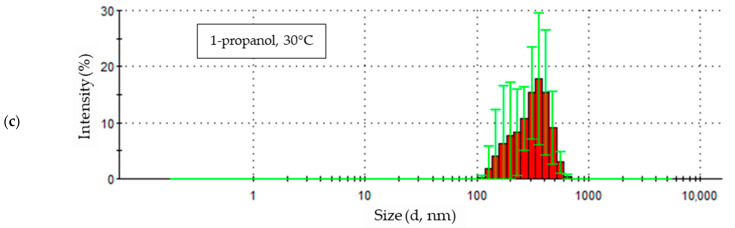
Statistical size distribution of ZnO nanoparticles synthesized in 0.05 M LiCl alcoholic solutions with 5% vol. H_2_O at 30 °C (five measurements) in (**a**) methanol, (**b**) ethanol, (**c**) 1-propanol.

In the sample of nanoparticles synthesized at 30 °C (Figure 7a), seven particle size fractions were identified based on five-fold measurement, ranging from particles ranging in size from approximately 340 nm to 880 nm. The largest quantitative share of particles with a size of about 480–590 nm, their intensity in the statistical distribution of particle sizes in the sample is about 27% and 27.5%, and this is how the share of this particle size in the entire sample can be estimated. The quantitative fractions of ZnO particles of other sizes are smaller, and in the case of the smallest and largest particles, their shares are significantly smaller than the most numerous particles.

Among the nanoparticles obtained in ethanol electrolyte at 30 °C (Figure 7b), twelve particle size fractions were identified. In the tested sample, smaller-sized particles, from about 120 nm to about 200 nm, and a small number with a diameter above 500 nm were recorded. The total quantitative share of the smallest and largest particles does not exceed 10% of the total marked particles. The remaining signals cover particles ranging in size between approximately 200 nm and 500 nm, reaching maximum intensities of 24% for particle sizes of 300–350 nm, representing almost half of all particles in the sample.

The statistical size distribution of ZnO nanoparticles obtained in a LiCl 1-propanol solution with the addition of 5% water by volume at a temperature of 30 °C is shown in Figure 7c. In terms of the number of particle fractions determined in the sample and their size distribution, it slightly resembles the distribution obtained for the ethanol solution (Figure 7b). The sample contained thirteen particle fractions. The most numerous of them are particles with a size of 356.2 nm, the intensity of which reaches 17.9%. The next two fractions, in terms of intensity values, are particles with sizes of 307.6 nm and 412.5 nm. Together with the fraction with the highest intensity, they constitute almost half (48.6%) of all particles in the sample. The share of larger particles is small and, based on the intensity value, amounts to 12.4%. The sizes of particles smaller than the dominant ones in terms of size are more diverse. They include seven fractions with sizes ranging from about 100 nm to about 250 nm, making up 38.8% of the particles in the sample.

### 3.3. Nanoparticles Electrochemical Synthesis at 40 °C

**Figure 8:**
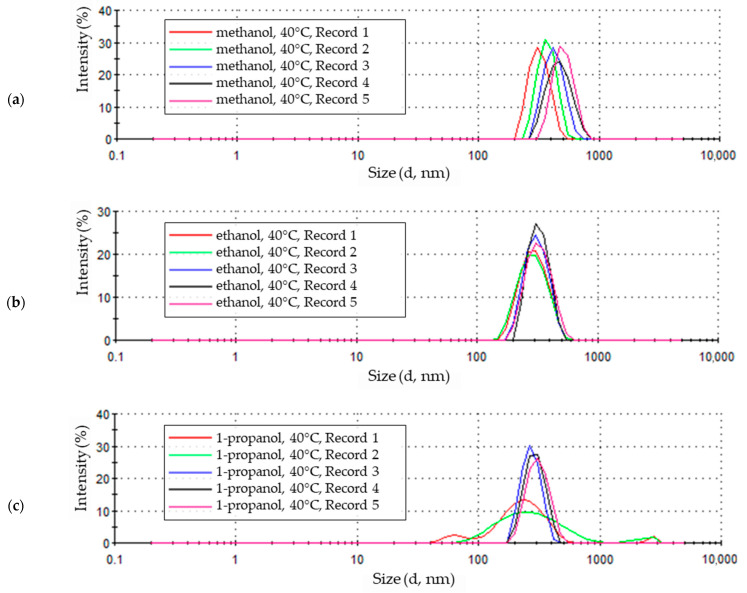
Zinc oxide nanoparticles size distribution by intensity in 0.05 M LiCl alcoholic solutions with 5% vol. H_2_O at 40 °C in (**a**) methanol (average particle size 566.1 nm), (**b**) ethanol (average particle size 323.1 nm), (**c**) 1-propanol (average particle size 285.4 nm).

The zinc oxide particle signals obtained from a solution with methanol solvent at 40 °C showed similar intensities in the first three measurements between approximately 29% and 31% (Figure 8a). The signal intensity of the fourth measurement is characterized by a lower intensity and a less distinct peak maximum at the level of approximately 23%. The maximum value of each subsequent measurement in the series is shifted towards increasing particle sizes. Also, the last measurement contained larger particle sizes for this sample, and its intensity again reached a significantly higher value, about 29%. This means that over time, the presence of particles of increasingly larger sizes and in comparable amounts was recorded for each subsequent measurement. The penultimate measurement in the series indicates the presence of fewer larger particles, and for the last (fifth) measurement of this sample, the number of larger particles increased again.

Increasing the synthesis temperature of zinc oxide nanoparticles to 40 °C changes the characteristics of the resulting synthesis product. The particle size distribution obtained at a temperature of 40 °C is shown in Figure 8b. The sample measurement results take the form of clearly developed peaks. The intensities of the first two reach comparable values of about 20% with particle sizes of about 290 nm. The next three peaks have even more developed maxima, indicating a particle size of approximately 300 nm, and therefore, slightly shifted towards larger particle sizes. The last three measurement peaks of the sample differ in their maximum intensity values, ranging between 22% and 27%. The highest value is achieved by the peak of the penultimate measurement and the lowest by the last peak. Changes in signal intensity values correspond to the ranges of particle sizes covered, indicated by the width of the peaks at the base. The first two recorded peaks of the particle size distribution, with the lowest intensity, also show the widest range of particle sizes, between about 140 and about 600 nm. The narrowest particle size range corresponds to the peak with the highest intensity and includes particles ranging in size from about 200 to about 530 nm. This proves quantitative changes in the most numerous particle size fractions.

In a sample containing zinc oxide nanoparticles produced in a 1-propanol solution at a temperature of 40 °C (Figure 8c), changes occurring in the colloidal solution over time during measurement are also visible. The signal of the first measurement covers a very wide range of particle sizes and shows as many as three intensity maxima: for a particle size of about 60 nm, it was about 3%; for a particle size of about 210 nm, it was about 13%; and about 2% for a particle size of about 2900 nm. In the second measurement, the maximum peak at the lowest particle size disappears; the other two remain, but their intensity decreases, especially in the case of the largest one. It covers a wide range of particle sizes from about 70 nm to about 1000 nm, with a maximum intensity of about 10% for particles of about 230 nm. The results of the third, fourth, and fifth sample measurements indicate the formation of particles in the solution that are more uniform in size than initially. The last three signals are well-shaped peaks with a similar but increasing particle size range from about 170 nm to 400–460 nm for the third and fourth measurements and to about 600 nm for the last measurement. The increasing range of particle sizes is accompanied by a shift in the maxima of subsequent peaks towards larger particle sizes, from about 250 nm for the third measurement to about 300 nm for the fourth and fifth measurements.

The value of the maximum peak intensity also decreased and became approximately 30% for the third, approximately 27% for the fourth, and approximately 25% for the last (fifth) measurement. As in the case of the sample of ZnO nanoparticles produced in a 1-propanol environment at 30 °C, the size of the identified particles also changed in the analyzed sample. However, the decreasing intensity of the peaks from the last three measurements and the shifting of their maximum indicates that the changes in the structure of the colloidal solution have not been completed and may continue after the measurement is completed.

**Figure 9:**
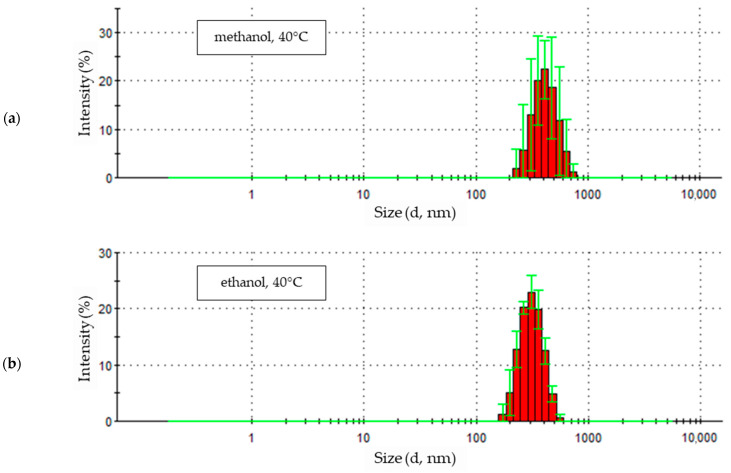

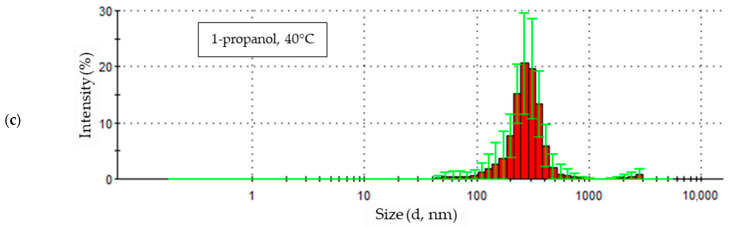
Statistical size distribution of ZnO nanoparticles synthesized in 0.05M LiCl alcoholic solutions with 5% vol. H_2_O at 40 °C (five measurements) in (**a**) methanol, (**b**) ethanol, (**c**) 1-propanol.

Particle samples obtained in methanol and ethanol solvents at 40 °C show similar particle size ranges. In both cases, nine particle fractions were recorded, ranging in size from just over 230 nm to about 740 nm for the methanol solution (Figure 9a) and from about 170 nm to about 600 nm for the ethanol solution (Figure 9b). The intensities of the most numerous fractions of recorded particles also reach similar maximum values. The maximum values reached 22.4% in the methanol solution and 23% in the ethanol solution, and this corresponds to a particle size of 412.5 nm in methanol and 307.6 nm in ethanol. Zinc oxide particles produced in methanol and ethanol solutions at 40 °C are more uniform in size. In both solutions, the three most numerous fractions constitute over 60% of all identified particles. The shares of the remaining factions, both larger and smaller than the most numerous ones, did not exceed 15%.

The statistical size distribution of particles formed in a 1-propanol solution with the addition of 5% water by volume at a temperature of 40 °C is shown in Figure 9c. The maximum intensity of 20.6% was achieved by the particle fraction of 265.2 nm. The particles in the sample were largely homogeneous. The four most abundant fractions in the sample made up almost 70% of the total particles in the sample and ranged in particle size from 229.3 nm to 356.2 nm. The share of smaller particles in the sample with 1-propanol solvent includes eleven fractions of particles with sizes between 45.64 nm and 198.0 nm. Except for three of them, with intensity values of 2.7%, 3.6%, and 7.6%, the rest did not exceed intensity values above 2%. The presence of particles larger than the most abundant ones in the sample is shaped similarly. In the size range above 356.2 nm up to 2780 nm, 11 particle fractions were also determined but did not reach intensity values above 1%.

### 3.4. Nanoparticles Electrochemical Synthesis at 50 °C

**Figure 10:**
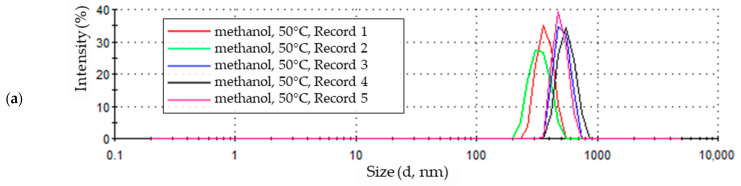

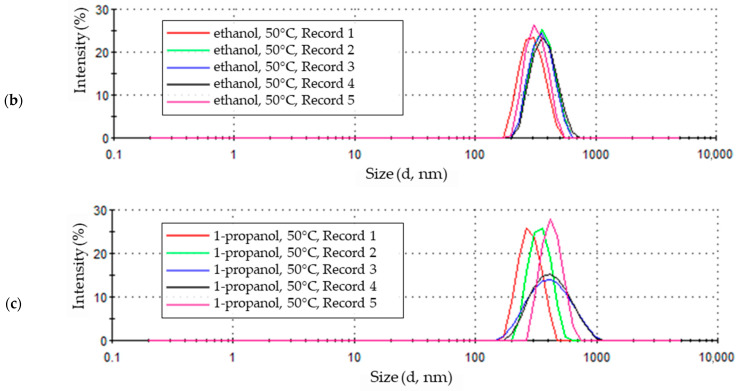
Zinc oxide nanoparticle size distribution by intensity in 0.05 M LiCl alcoholic solutions with 5% vol. H_2_O at 50 °C in (**a**) methanol (average particle size 616.3 nm), (**b**) ethanol (average particle size 371.2 nm), (**c**) 1-propanol (average particle size 415.7 nm).

The highest temperature at which the electrochemical synthesis of zinc oxide nanoparticles was carried out was 50 °C. The particle size distribution in a methanol solution with the addition of 5% water by volume is shown in Figure 10a. The first of the recorded signals reached an intensity of approximately 35%, and its maximum corresponds to particles with a diameter of approximately 340 nm. The second measurement shows a reduction in signal intensity, approximately 28%, and a shift of the less developed maximum value towards smaller particle sizes—approximately 300 nm. The last three signals are clustered in the particle size range with a diameter between approximately 340 and 900 nm, with prominent maxima whose intensity increases from approximately 34% to 39% for the last measurement. This means an initial decrease, then an increase and stabilization of particle size at the end of the test.

Zinc oxide nanoparticles formed in ethanol solution at 50 °C show the greatest size uniformity compared to others synthesized at this temperature. This is evidenced by the similar values of the maximum intensity values and the narrow and slightly shifted peak bases of all five sample measurements (Figure 10b). The peaks of the measurement signals (maxima) range in particle size from approximately 300 nm to 380 nm.

The size distribution of zinc oxide nanoparticles formed in a 0.05 M 1-propanol LiCl solution with the addition of 5% water by volume, similar to the previous samples, indicates changes occurring in the structure of the colloidal solution (Figure 10c). Changes occurred during sample testing between subsequent measurements, but they were of a different nature. Initially, two peaks with similar intensities of approximately 25% were recorded, but with the maximum intensity, the values shifted relative to each other. For the first peak, the position of the maximum corresponds to a particle size of approximately 260 nm; for the second peak, it is approximately 350 nm. The first two measurement peaks also have a similar width at the base, corresponding to the overall range of particle sizes identified in a given measurement. They take values between about 50 nm and 500 nm for the first measurement and from about 200 nm to about 600 nm for the second measurement. The structure of the colloidal solution changed between the second and third measurements. The results of the third and fourth measurements were broad, less defined peaks with mild peaks of approximately 15% and 16%, with a particle size of approximately 400 nm. The lower maximum intensity of these peaks corresponds to a much wider range of recorded particle sizes. In the case of the third and fourth peaks, these ranges are from about 60–70 nm to about 1000 nm.

The last of the five sample measurements did not show a decreasing intensity trend that might be expected based on the earlier peaks. The result of the last measurement, again as in the case of the first and second measurements of the sample, formed a clearly shaped peak with a maximum intensity of about 28% and a particle size of about 450 nm. The base of the peak covers particle sizes between about 250 nm and about 800 nm and is, therefore, narrower than the third and fourth measurements, with the peak maximum slightly shifted towards larger particle sizes. The shift in the maximum of each of the five subsequent measurement peaks indicates changes occurring in the solution, resulting in the formation of particles of increasingly larger sizes. In the first stage of the test (first measurement), the smallest particles of relatively uniform size were present in the sample. In the second measurement, particles with a small size range were also recorded, but the size of all particles identified in the sample was slightly larger. This size distribution is the result of the ultrasonic treatment at the sample preparation stage before measurement, which causes the breakdown of particle agglomerates. The third and fourth results indicate changes occurring in the sample volume. Broad peaks with a gentle maximum indicate the presence of particles of very different sizes in the sample. The result of the last measurement is a peak indicating the end of the changes represented by the peaks from the third and fourth measurements and the re-appearance of larger particles with more uniform sizes in the solution.

**Figure 11:**
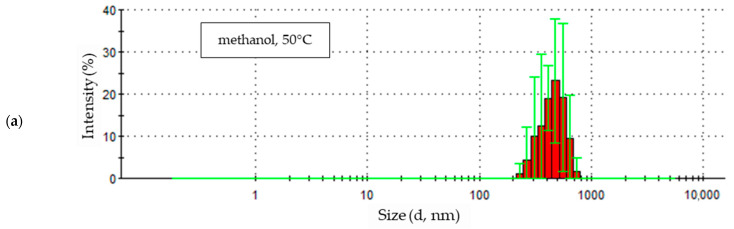

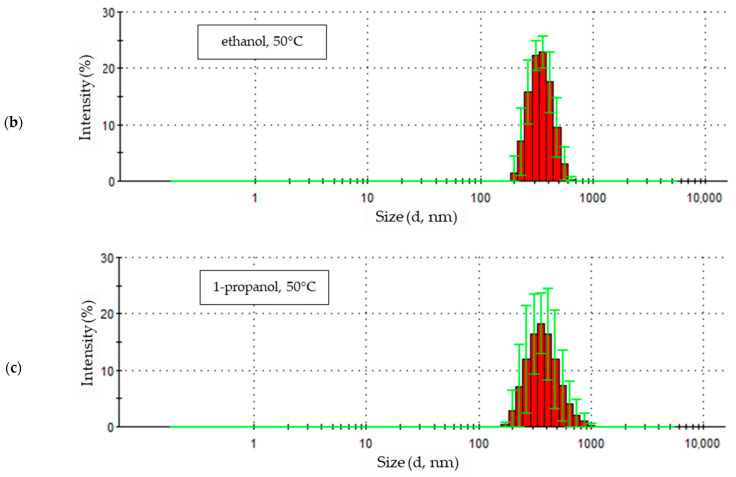
Statistical size distribution of ZnO nanoparticles synthesized in 0.05 M LiCl alcoholic solutions with 5% vol. H_2_O at 50 °C (five measurements) in (**a**) methanol, (**b**) ethanol, (**c**) 1-propanol.

The graphs in Figure 11 show the statistical size distribution of zinc oxide particles synthesized in a 0.05 M LiCl solution in alcoholic solvents with the addition of 5% water by volume at a temperature of 50 °C.

In the sample of nanoparticles obtained in methanol, nine size fractions of the particles being formed were recorded as a result of the study (Figure 11a). The most numerous fraction contained particles with a size of 477.7 nm; their share was 23.1% and together with the other two fractions with the highest intensity—19.0% for particles with a size of 412.5 nm and 19.1% for a particle size of 553.7 nm—constitute 61.2% of all particles in the sample. Larger particles were less numerous than smaller particles, but their sizes were less diverse. The two largest size fractions accounted for 10.9% of the total particles. The smallest particles in the sample varied in size, with four fractions, and their share in the total number of particles was 27.9%.

The intensity maximum for the particle size distribution produced in the ethanol solution at 50 °C reached a value of 22.9% for particles with a particle size of 356.2 nm (Figure 11b). The second most abundant particle fraction had a signal intensity of 22.3% for particles with a size of 307.6 nm; together, the two most abundant sizes constitute over 45% of all particles present in the sample. In addition to the two most abundant sizes, three fractions of smaller particles and four fractions of larger particles were identified in the sample. The larger four particle fractions constitute 30.6% of the total particles in the sample, and smaller particles, among which there are three fractions, total abundance is 24.2%.

The sample obtained in a solution with a 1-propanol solvent at a temperature of 50 °C (similar to the process temperature of 30 °C) contained the largest number of particles with a size of 356.6 nm, for which the measurement intensity was 18.3% (Figure 11c). The next two results with the highest intensity were for particles of 307.6 nm and 412.5 nm, and their values were 16.5% and 16.4%, respectively. The three most numerous fractions in the sample constituted more than half (51.2%) of the number of particles identified in the tested solution. The remaining part consisted almost equally of particles of smaller and larger sizes. Smaller particles were more homogeneous and were divided into four fractions whose quantitative share of particles was 22.3%, while among the larger particles, there are six fractions, the sum of which is equal to 26.6% of the total number of particles.

## 4. Discussion

### 4.1. Nanoparticles Electrochemical Synthesis at 20 °C

ZnO (nano)particles produced at 20 °C in alcohol solutions of 0.05 M LiCl with the addition of 5% water by volume, regardless of the solvent used, methanol, ethanol, or 1-propanol, show significant differences in size. In the initial phase of measurement, particles of the smallest sizes were recorded, as indicated by the high intensity of the first peaks. The narrow width at the base of these peaks suggests the presence of particles that are relatively uniform in size. Over time, in each solvent environment, particles with greater variation in size and larger particles were recorded in the samples. This indicates that particles tend to cluster together and form agglomerates as they combine and grow in size throughout the measurement.

### 4.2. Nanoparticles Electrochemical Synthesis at 30 °C

Increasing the temperature of ZnO nanoparticle synthesis to 30 °C, without changing any other parameters of the reaction environment, resulted in the opposite phenomenon to that observed at 20 °C. At 30 °C in 0.05 M LiCl solutions with methanol and ethanol solvents with the addition of 5% water by volume, in the first two measurements for the sample, peaks with lower intensity and smaller particle sizes were recorded.

In the case of the 1-propanol-based electrolyte, at the beginning of sample testing, the presence of the smallest particles was recorded for this sample, which was highly uniform in size (Figure 6). The next two measurements performed on the 1-propanol-based sample gave results similar to those observed in the initial phase of the measurement for the sample with ethanol solution. For both samples, in ethanol and 1-propanol, the second signal for the series of five had the lowest intensity. The intensity of the peaks decreased, and the size range of identified particles widened.

The results of the last two measurements for the 1-propanol medium and the last three measurements for methanol and ethanol solutions clearly indicate, in all cases, the formation of larger particles in the tested solutions, with a high degree of size uniformity.

Therefore, the statistical distributions of particle sizes obtained at 30 °C (Figure 7) in all types of solvents, except 1-propanol, showed a small scatter of particle sizes. The graphs took the form of columns of high intensity with a small number of fractions.

### 4.3. Nanoparticles Electrochemical Synthesis at 40 °C

A further increase in the process temperature, up to 40 °C, caused the ZnO nanoparticles produced at this temperature to undergo structural changes during the study. In all solutions, the particle size increased during measurement (Figure 8).

The smallest changes in the size of ZnO particles were observed in the ethanol solution (Figure 8b). The slight increase in particle size was accompanied by a regular increase in maximum intensity until the penultimate measurement. The maximum intensity decreased in the last measurement. A similar behavior was observed in the 1-propanol solution (Figure 8c). The maximum intensity value and high uniformity in particle size were observed in the third measurement of the sample; in the fourth and fifth measurements, the intensity of the peaks decreased successively. Additionally, in the 1-propanol electrolyte, the first two recorded signals clearly differed from the others, taking much lower intensity values and covering a very wide range of particle sizes. This proves the presence of particles of very different sizes at the beginning of the measurement. Despite the fact that the sample preparation, using an ultrasonic scrubber, was intended to break down the particle agglomerates formed in the sample.

The results recorded for a sample from a methanol solution indicate that the particles formed in the solution increased in size over the course of the test (Figure 8a). The maximum signal intensity fluctuated within a small range. This suggests that the particles grow progressively larger while maintaining high uniformity.

The statistical distribution of particle sizes obtained in alcohol solutions at 40 °C (Figure 9) indicates that the particles were characterized by high homogeneity. This is proven by the high values of intensity maxima and the limited number of determined particle size fractions in all tested solutions. Even in the case of a 1-propanol solution, despite the initial presence of very small and very large particles, the particle size stabilized in the final phase of measurement.

### 4.4. Nanoparticles Electrochemical Synthesis at 50 °C

The highest temperature used for the synthesis of zinc oxide nanoparticles in alcohol solutions of 0.05 M LiCl, with the addition of 5% water by volume, was 50 °C.

Initially, particles of uniform and smallest sizes, except for the methanol solution, were recorded in all solutions (Figure 10). With each subsequent measurement, particle size increased in all media. In methanol and ethanol solutions, the formation of the largest particles was recorded for the third and fourth runs in the series of five. Then, in the final measurement results for these samples, a reduction in particle size was observed while the signal intensity increased (Figure 10a,b). The third and fourth measurements for the 1-propanol solution recorded the lowest intensity values. The last measurement for this sample, similar to samples in other solvents, had the highest intensity, with particle sizes remaining constant (Figure 10c).

The sample of particles obtained in ethanol solution was the most uniform in terms of particle size (Figure 10b). Homogeneous particles were present in the methanol solution, even though their sizes changed during measurement (Figure 10a). In a sample of particles obtained in a 1-propanol solution, initially, homogeneous particles (first and second measurements) transitioned into particles with a wide range of sizes. Eventually, in the final part of the measurement, particles regained homogeneity (Figure 10c).

For all analyzed environments, the particle sizes recorded at the end of the study were the most uniform.

### 4.5. Discussion Summary

The DLS method, commonly used to measure the size of particles or nanoparticles in colloidal solutions, proved ineffective for the tested solutions. Structure changes occurred in the colloidal solutions during particle size measurements. ZnO particles within the solution tended to concentrate and agglomerate. Due to the change in the size of the particles, the obtained measurement results cannot be considered conclusive.

Based on the measurements performed, it is not possible to consistently determine the size of zinc oxide particles. It is only possible to approximately estimate the initial particle size in the solution after the agglomerates have broken down using an ultrasonic scrubber. Due to structural changes occurring within the sample, in the time it takes to place the sample in the device and perform the first measurement in a series of five, an approximate result of the initial particle size can be obtained.

Changes occurring in the particle structure during measurement also prevent the determination of the final particle size. After completing the test, there is no certainty that the duration of the series of measurements corresponds to the time in which structure changes occur in the solution. To determine this, it would be necessary to conduct comparative tests using samples that were not sonically crushed.

The use of the DLS method using the Zetasizer Nano ZS device to measure the size of zinc oxide nanoparticles allowed for determining changes in particle size that occur during measurement.

As a result of measurements performed on the size of zinc oxide particles in the tested solutions, the average sizes of the recorded particles reached values exceeding 500–600 nm. Hence, they are referred to as particles in the text instead of nanoparticles, in accordance with the definitions of nanomaterials found in the sources, covering structures with sizes from 1 to 100 nm [4,6,39,40].

The sizes of particles identified in the tested colloidal solutions using the DLS method are significantly larger than those shown in Figure 3. However, it should be noted that the ZnO nanoparticles visible in TEM images were specially prepared. The sample material consisted of powders obtained after rinsing and evaporating the solvent. Unlike ZnO particles forming a colloidal suspension in the state during synthesis, they were free from contamination with electrolyte components.

Particles forming a colloidal suspension can agglomerate due to various factors, including their structure, properties, or the influence of external conditions [41,42].

One of the fundamental features of nanomaterials is a very large specific surface area [43]. It is a measure of the surface area of a substance in relation to its volume, which increases as the particle size decreases. The highly developed specific surface area of the nanomaterial causes an increase in surface energy. To minimize this energy, the particles tend to reduce the specific surface area by forming agglomerates. Larger structures with lower energy are more thermodynamically stable.

Another feature that favors the agglomeration of nanoparticles is the structure of the ZnO particle. The difference in electronegativity between oxygen atoms O (E = 3.44) and zinc atoms Zn (E = 1.65 according to Pauling) is ΔE = 1.79. This value classifies the bond in the ZnO molecule as an ionic bond [44]. The bond in the zinc oxide molecule, although formally it should be an ionic bond, also has the features of a covalent bond. The charge is not evenly distributed in the molecule, so it has a dipole moment, i.e., it is polar. This feature makes the nanometric ZnO particles in the solution capable of adsorbing ions or other polar molecules. The main components of the tested solutions were alcohols and water–polar compounds. The ability to attract and accumulate ions or whole molecules certainly increases the size of ZnO nanoparticle agglomerates. This agglomerate can take the form of micelles, spherical structures with an ordered distribution of charges. This suggests that in the tested solutions, the structures with identical charges may be present. These structures could be capable of repulsing each other preventing agglomeration. However, this balance can be disrupted by the accumulation of chloride anions on the surface of agglomerates. These anions originate from the dissociation of lithium chloride in solution. Uneven adsorption of chloride ions can lead to charge equalization, causing the particle clusters to lose their repulsive forces and enabling further agglomeration. This may be counteracted by the additional accumulation on the surface of agglomerates of chloride anions present in the solution originating from dissociated lithium chloride. Uneven adsorption of chloride ions may lead to charge equalization, which causes the particle clusters to lose the ability to repel each other.

Physical phenomena occurring on the surface of particle agglomerates may influence the value of the zeta potential (ζ). The value of the electrokinetic potential at the particle–electrolyte interface is also one of the factors determining the formation of particle clusters. High zeta potential values (>±30 mV) can stabilize nanoparticle suspensions through electrostatic repulsion [42,45]. The formation of large particle clusters observed in the tested solutions, particularly at 20 °C, may indicate low zeta potential values for the particles formed in those environments. This is supported by the results of zeta potential measurements carried out for nanoparticle samples obtained at 20 °C. Zeta potential values for 0.05 M LiCl solutions with the addition of 5% vol. water for methanol, ethanol, and 1-propanol solvents were: −9.45 ± 0.75 mV, −9.42 ± mV, and −13.65 ± 1.25 mV, respectively.

The formation of particle clusters also depends on the type of alcohol solvent, which constitutes a significant part of the volume of the analyzed solutions. As the length of the carbon chain in the alcohol molecule increases, the degree of dissociation in these solutions decreases.

A comparison of the results of the presented research supports certain previously observed relationships.

The sizes of the formed ZnO nanoparticles decrease as the length of the alcohol carbon chain increases. In previous studies [34,35], this relationship was observed for nanoparticles produced at 20 °C.

**Figure 12:**
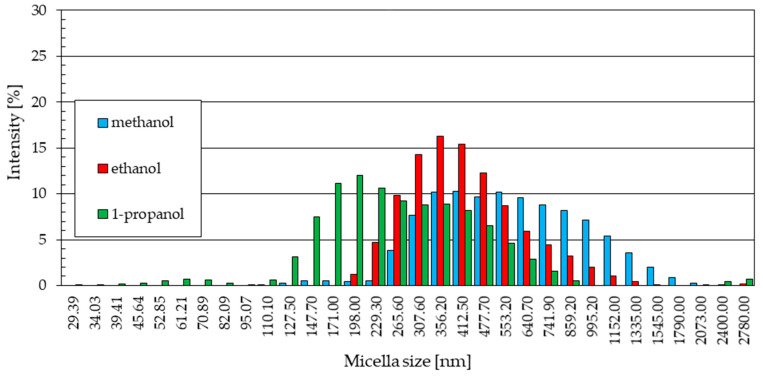
ZnO nanoparticle size distribution in 20 °C.

The particles present in the methanol solution were the largest, and the particles in the 1-propanol solution were the smallest (Figure 12). The size of the particles formed in the ethanol solution was in between. The intensity of samples from the ethanol solution was higher than the others. This is confirmed by the results of previous studies [34,35], determining nanoparticles produced in ethanol as the most uniform in terms of size. This is confirmed by the highest intensity values for the ethanol sample.

The sizes of ZnO particles produced in the 1-propanol electrolyte were mostly the smallest (Figure 12). The problem in the case of the synthesis of zinc oxide nanoparticles in a 1-propanol medium is the purity of the product. As a result of the synthesis process, in addition to ZnO, other phases were also formed, such as zinc hydroxide, chloride salts, and hydroxysalts, as well as complex compounds. It is likely that these impurities contribute to the presence of small amounts of fractions with both very small and very large particle sizes in the size distribution graphs.

Research by other authors [34,35] confirms that nanoparticles obtained using a methanol solvent have a wide range of sizes. In the methanol solution, the formation of nanoparticles occurred with the greatest intensity, but their size was random.

Previous studies conducted at 20 °C [34,35] showed a relationship between the size of the particles formed and the type of solvent.

Moletica et al. [46,47] confirmed the dependence of the size and shape of the ZnO nanoparticles formed on the reaction environment. Despite using a different method to synthesize zinc oxide nanoparticles, the researchers proved that the shape and size of ZnO nanoparticles depend on the type of alcohol used in the synthesis. For successive alcohols, they observed a change in shape from spherical, through polyhedral, to rod-like structures, with increasing carbon chain length in the alcohol molecule.

**Figure 13:**
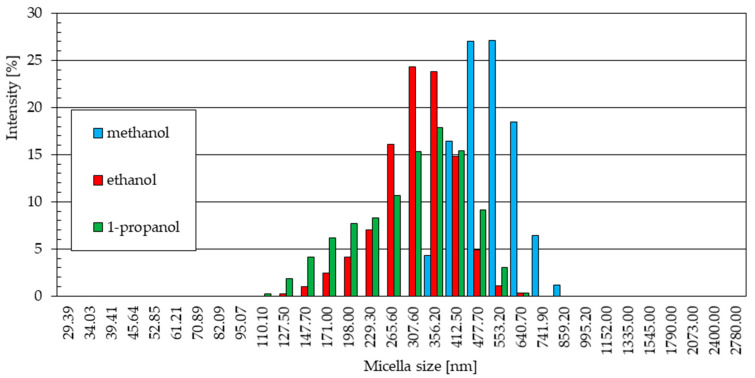
ZnO nanoparticle size distribution in 30 °C.

**Figure 14:**
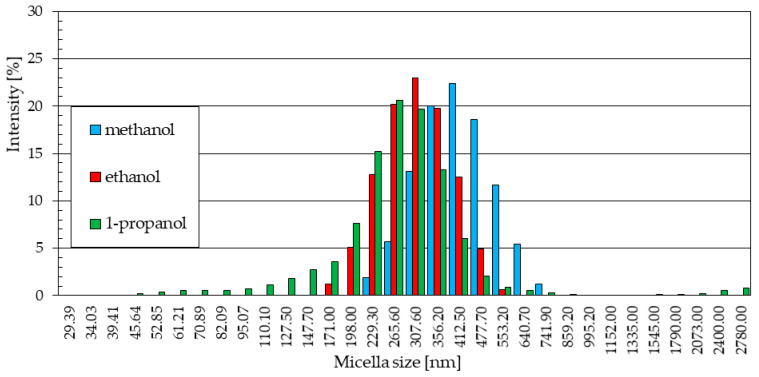
ZnO nanoparticle size distribution in 40 °C.

**Figure 15:**
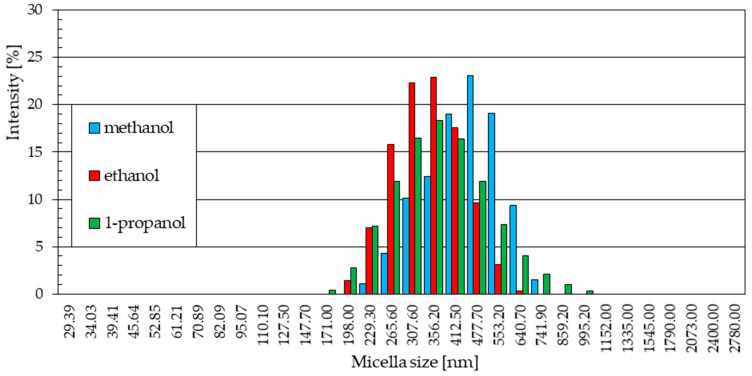
ZnO nanoparticle size distribution in 50 °C.

Our research confirmed this relationship (Figure 12). The average particle size increased as the length of the carbon chain in alcohol increased. We also showed that this dependence occurs at synthesis temperatures of 30 °C and 40 °C (Figure 13 and Figure 14). However, at a temperature of 50 °C, this relationship no longer applies (Figure 15).

At the highest temperature used for the synthesis of ZnO nanoparticles (50 °C), the average size of the particles formed in methanol was the largest, while the size of the particles in ethanol was smaller than in 1-propanol (Figure 15).

Also noteworthy is the greatest uniformity of sizes of ZnO nanoparticles synthesized at 50 °C, regardless of the solvent used. In all of the tested solutions, the size range of the particles formed was remarkably similar. 

The shares of all size fractions of zinc oxide nanoparticles synthesized in alcohol solutions, along with standard deviation values, depending on the temperature of the synthesis process, are summarized in the tables in Appendix A.

**Figure 16:**
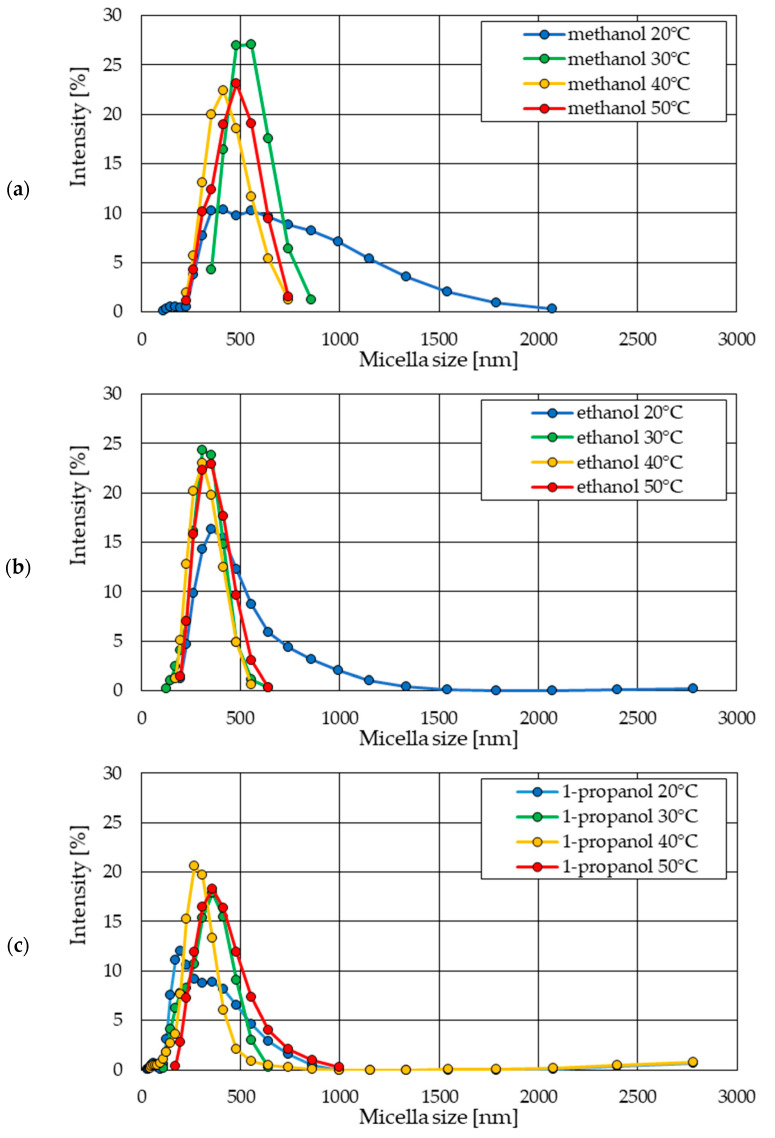
Comparison of statistical size distributions of ZnO nanoparticles synthesized in solutions with alcohol solvents at different temperatures: (**a**) methanol; (**b**) ethanol; (**c**) 1-propanol.

By comparing the results of the size distribution of the ZnO nanoparticles obtained in each of the alcohol solvents used, considering the synthesis temperature, it is possible to present the influence of alcohol on the form of the final product of the synthesis process (Figure 16a–c).

The smallest impact of the increased process temperature on the size and size distribution of the formed particles was observed in the ethanol environment (Figure 16b). The particles obtained at elevated temperatures were the most homogeneous. A similar range in particle size distribution and maximum intensity also means the greatest stability of the tested suspension.

Particle size distribution charts for methanol and 1-propanol solutions, depending on temperature, do not show such uniformity, as was observed in the case of the ethanol solution (Figure 16a–c).

In samples from all types of solvents, the smallest particles were obtained at 40 °C. This is especially visible in methanol and 1-propanol solutions. The formation of the largest particles took place in the methanol solvent at 30 °C and in the 1-propanol solvent at temperatures of 30 °C and 50 °C, similar to the ethanol solvent (Figure 16a–c).

The results of basic research demonstrate that the morphology of zinc oxide nanoparticles synthesized via electrochemical methods is influenced by multiple factors, including the type of alcohol solvent used and the temperature of the process. Temperature emerges as a critical parameter alongside the alcohol type, salt concentration in the solution, and water addition. This highlights the need for a more precise understanding of the role of temperature, as well as the changes in the zeta potential of agglomerating particles during synthesis.

Despite the need to optimize several parameters, this method of synthesizing zinc oxide nanoparticles in alcoholic salt solutions with water addition is notably straightforward. It avoids the need for stringent conditions such as high temperature, elevated pressure, or tightly sealed reaction systems, making it a practical and accessible approach.

The electrochemical synthesis of zinc oxide nanoparticles is environmentally friendly. Its low production cost, simplicity, and lack of environmental burden make it a strong contender against traditional nanoparticle production methods such as the sol-gel method or co-precipitation. Moreover, like the microfluidic method, it represents an innovative and viable alternative for nanoparticle synthesis [48].

However, this method is not without challenges. The primary drawback is the contamination of nanoparticles with chloride ions from the reaction medium. This issue can be addressed by rinsing the pre-decanted nanoparticle suspension with the same alcohol used during synthesis, effectively removing the chloride ions.

A more significant challenge lies in scaling up the process for industrial applications and accurately determining production yields. Addressing these issues will be crucial for the widespread adoption of the electrochemical method in nanoparticle synthesis.

Refining the electrochemical synthesis method for obtaining nanoparticles is highly significant, as improved control over the synthesis process—including the ability to regulate nanoparticle shape and size—directly impacts their application potential. Zinc oxide nanoparticles, with their unique properties, continue to gain attention as a versatile material with expanding applications.

In addition to their well-established uses, ongoing research is uncovering new ways to leverage their properties in various fields. Emerging applications include electronics [49], medicine [48], and biomedicine [50], demonstrating the material’s growing importance across diverse industries.

## Data Availability

The original contributions presented in this study are included in the article. Further inquiries can be directed to the corresponding authors.

## Appendix A

**Table A1:** Summary of ZnO particle size shares with standard deviation values recorded in alcoholic solutions of 0.05 M LiCl with 5% water by volume, synthesized at 20 °C.

Particles Diameter	Fraction Share in Methanol	Standard Deviation	Fraction Share in Ethanol	Standard Deviation	Fraction Share in 1-Propanol	Standard Deviation
[nm]	[%]	[%]	[%]	[%]	[%]	[%]
29.39					0.10	0.10
34.03					0.10	0.30
39.41					0.20	0.40
45.64					0.30	0.50
52.85					0.50	0.70
61.21					0.70	1.00
70.89					0.60	0.90
82.09					0.30	0.50
95.07					0.10	0.10
110.10	0.10	0.30			0.60	1.40
127.50	0.30	0.70			3.10	6.60
147.70	0.50	1.00			7.50	11.40
171.00	0.50	1.10			11.10	13.60
198.00	0.40	0.90	1.20	1.80	12.00	12.00
229.30	0.50	0.60	4.70	4.70	10.60	8.70
265.60	3.80	8.10	9.80	7.30	9.20	7.20
307.60	7.70	14.30	14.30	8.10	8.80	8.40
356.20	10.20	13.20	16.30	6.60	8.90	8.50
412.50	10.30	9.30	15.40	3.50	8.20	7.60
477.70	9.70	9.40	12.30	3.20	6.50	6.80
553.20	10.20	7.10	8.70	5.80	4.60	6.00
640.70	9.60	6.10	5.90	6.80	2.90	4.40
741.90	8.80	7.50	4.40	6.00	1.60	2.40
859.20	8.20	7.50	3.20	4.30	0.50	0.90
995.20	7.10	6.50	2.00	2.80		
1152.00	5.40	5.10	1.00	1.50		
1335.00	3.60	3.70	0.40	0.70		
1545.00	2.00	2.40	0.10	0.20		
1790.00	0.90	1.20				
2073.00	0.30	0.40			0.10	0.30
2400.00			0.10	0.20	0.40	0.80
2780.00			0.20	0.50	0.70	1.50

**Table A2:** Summary of ZnO particle size shares with standard deviation values recorded in alcoholic solutions of 0.05 M LiCl with 5% water by volume, synthesized at 30 °C.

Particles Diameter	Fraction Share in Methanol	Standard Deviation	Fraction Share in Ethanol	Standard Deviation	Fraction Share in 1-Propanol	Standard Deviation
[nm]	[%]	[%]	[%]	[%]	[%]	[%]
29.39						
34.03						
39.41						
45.64						
52.85						
61.21						
70.89						
82.09						
95.07						
110.10					0.20	0.40
127.50			0.20	0.40	1.80	4.00
147.70			1.00	1.60	4.10	8.30
171.00			2.40	3.60	6.20	10.50
198.00			4.10	5.80	7.70	9.50
229.30			7.00	6.90	8.30	7.70
265.60			16.10	5.50	10.70	5.70
307.60			24.30	8.60	15.30	8.30
356.20	4.30	2.60	23.80	9.10	17.90	11.80
412.50	16.40	6.10	14.80	7.00	15.40	11.10
477.70	27.00	5.90	4.90	5.20	9.10	6.50
553.20	27.10	3.10	1.10	2.00	3.00	1.90
640.70	18.50	6.10	0.30	0.70	0.30	0.50
741.90	6.40	6.00				
859.20	1.20	1.60				
995.20						
1152.00						
1335.00						
1545.00						
1790.00						
2073.00						
2400.00						
2780.00						

**Table A3:** Summary of ZnO particle size shares with standard deviation values recorded in alcoholic solutions of 0.05 M LiCl with 5% water by volume, synthesized at 40 °C.

Particles Diameter	Fraction Share in Methanol	Standard Deviation	Fraction Share in Ethanol	Standard Deviation	Fraction Share in 1-Propanol	Standard Deviation
[nm]	[%]	[%]	[%]	[%]	[%]	[%]
29.39						
34.03						
39.41						
45.64					0.20	0.40
52.85					0.40	0.80
61.21					0.50	1.00
70.89					0.50	0.90
82.09					0.50	0.70
95.07					0.70	1.00
110.10					1.10	1.60
127.50					1.80	2.60
147.70					2.70	3.70
171.00			1.20	1.70	3.60	4.90
198.00			5.10	4.10	7.60	3.80
229.30	1.90	4.10	12.80	3.20	15.20	5.30
265.60	5.70	9.50	20.20	1.10	20.60	8.90
307.60	13.10	11.60	23.00	3.00	19.70	8.80
356.20	20.00	9.20	19.80	3.40	13.30	5.90
412.50	22.40	6.10	12.50	2.30	6.00	3.80
477.70	18.60	10.50	4.90	1.30	2.10	2.30
553.20	11.70	11.20	0.60	0.70	0.90	1.80
640.70	5.40	6.60			0.50	1.20
741.90	1.20	1.70			0.30	0.70
859.20					0.10	0.30
995.20						
1152.00						
1335.00						
1545.00					0.10	0.10
1790.00					0.10	0.30
2073.00					0.20	0.50
2400.00					0.50	0.70
2780.00					0.80	1.10

**Table A4:** Summary of ZnO particle size shares with standard deviation values recorded in alcoholic solutions of 0.05 M LiCl with 5% water by volume, synthesized at 50 °C.

Particles Diameter	Fraction Share in Methanol	Standard Deviation	Fraction Share in Ethanol	Standard Deviation	Fraction Share in 1-Propanol	Standard Deviation
[nm]	[%]	[%]	[%]	[%]	[%]	[%]
29.39						
34.03						
39.41						
45.64						
52.85						
61.21						
70.89						
82.09						
95.07						
110.10						
127.50						
147.70						
171.00					0.40	0.50
198.00			1.40	2.90	2.80	3.70
229.30	1.10	2.40	7.00	5.90	7.20	7.40
265.60	4.30	7.80	15.80	5.70	11.90	9.50
307.60	10.10	13.90	22.30	2.60	16.50	7.10
356.20	12.40	17.10	22.90	2.80	18.30	5.40
412.50	19.00	7.80	17.60	5.30	16.40	8.10
477.70	23.10	14.80	9.60	5.30	11.90	8.70
553.20	19.10	17.50	310	3.00	7.30	6.30
640.70	9.40	10.50	0.30	0.60	4.00	4.20
741.90	1.50	3.40			2.10	2.80
859.20					1.00	1.40
995.20					0.30	0.40
1152.00						
1335.00						
1545.00						
1790.00						
2073.00						
2400.00						
2780.00						
